# Effect of gender on spect myocardial perfusion imaging results in Egypt

**DOI:** 10.1186/s43044-024-00560-9

**Published:** 2024-09-12

**Authors:** Taghreed Abdel-Rahman Ahmed, Ahmed Al-Habbaa, Mona Naiem, Naglaa Mokhtar, Fatma Elhady

**Affiliations:** 1 Cardiology Department, Faculty of Medicine for Girls, Al-Azhar University, Cairo, Egypt; 2 Cardiology Department, Faculty of Medicine, Al-Azhar University, Cairo, Egypt

## Abstract

**Background:**

Globally, Ischemic heart disease (IHD) is considered a leading cause of mortality and morbidity affecting men than women. The more the population ages, the more the prevalence. There was a concern about improper referral of women to MPI testing. We aimed to study if there a gender effect on the results of MPI studies and if this could have an impact on future referral or investigation selection for diagnosis of IHD as a general or specially in women.

**Results:**

Female gender represented 266 (60%) while male represented in 177 (40%). Males demonstrated significantly higher age (55 ± 10 vs. 49 ± 9, *P* < .0001), weight (85 ± 11 vs. 83 ± 13, *P* = 0.006), height (166 ± 4 vs. 165 ± 4, *P* = 0.02), and smoking (35% vs. 0%, *P* < 0.001) than females. Male gender was associated with ten times increased risk of positive MPI (OR = 10, 95% CI = 5.348–18.868, *P* < 0.001). Diabetes was associated with an increased risk of positive MPI (OR = 1.82, 95% CI = 1.052–3.148, *P* = 0.032).

**Conclusions:**

Positive MPI test are more common in males. Female patients with positive MPI were younger in age than male patients. Diabetes mellitus and age are traditional strong predictors for the presence of positive MPI test.

## Background

Globally, Ischemic heart disease (IHD) is considered a leading cause of mortality and morbidity affecting men than women [1]. The age standardized IHD prevalence in Egypt was 5.89% in 2019 with a nearly 5% increase from previous values reported in 2005 [2]. The more the population ages, the more is the prevalence [3].

The diagnosis of IHD needs careful history taking and risk profile assessment [4]. The pretest probability is the determinant of the choice of the diagnostic investigation [5]. Invasive coronary angiography is still the standard method for diagnosis of epicardial coronary artery stenoses, but it cannot assess the physiological effect of such lesions and carries many hazards [6]. Myocardial perfusion imaging (MPI) is one of the non-invasive tools to diagnose IHD [7]. MPI test could identify patients who may benefit from revascularization [8]. There was a concern about improper referral of women to MPI testing [9]. We aimed to study if there a gender effect on the results of MPI studies and if this could have an impact on future referral or investigation selection for diagnosis of IHD as a general or specially in women.

## Methods

The current retrospective observational study included 443 patients who were candidates for MPI throughout the period from January 2021 to October 2023.

### Ethical consent

Following the ethical perspectives of Helsinki Declaration, the study was approved by ethical committee, Al-Azhar University. A written informed consents were obtained from all eligible patients.

### Inclusion criteria

Symptomatic male or female patients with unexplained dyspnea, typical chest pain and fatigue referred to MPI unit to investigate coronary artery disease. All are adult (age > 18 years old) and able to exercise on treadmill.

### Exclusion criteria

1) Atrial fibrillation and other arrhythmias interfering with gated SPECT results, 2) LBBB in ECG, 3) History of chronic obstructive airway disease, 4) pulmonary hypertension, 5) Pregnancy or breast-feeding and 6) Morbid obesity and weight more than 120 kg that is beyond the table tolerability.

Detailed history was taken for HTN, DM, smoking, dyslipidemia, and FH of premature CAD), Baseline measurements for body mass index (BMI) were reported. Chest pain and dyspnea were also reported. Complete chest and cardiac examination with recording HR, and BP was done as well as surface ECG.

Myocardial perfusion SPECT studies: Stress protocol: treadmill exercising stress test was done for all patients [10].

### Myocardial perfusion imaging (MPI)

In accordance with a two-day procedure, a gated-SPECT MPI was performed for each patient. Patients with diaphragmatic and/or breast attenuation for females were corrected by prone position. Supine images were captured with a dual-head (Philips Cardio-MD system) camera equipped with low-energy, high-resolution collimators. Each radionuclide image and the associated data were processed using standard procedures.

99mTc SestaMIBI, the standard tracer used in our laboratory, was employed in every case. Myocardial perfusions were calculated as the relative percent absorption of tracers in each of the 17 segments using the standard paradigm. Nuclear cardiology specialists used both quantitative and qualitative data analysis to interpret each MPI scan.

The tracer uptake for each segment was assigned a score, which was as follows: (0) normal uptake, (1) mildly reduced, (2) significantly reduced, (3) severely reduced, and (4) nonexistent. The scores of the 17 segments of the stress and rest images were added to get the summed rest score (SRS) and summed stress score (SSS), respectively. The SRS was subtracted from the SSS to get the summed difference score (SDS). Studies were categorized as abnormal (SSS > 4) or normal (SSS < 4) depending on the results. To obtain LVEF, quantified gated SPECT software processed the gated images. Independent studies have reported on other high-risk perfusion scanning signs, including abnormal regional and global wall movement anomalies, transient ischemic dilatation (TID), and higher lung heart ratio (LHR) [11].

### Statistical analysis

For the statistical study, IBM, Armonk, New York, USA, SPSS version 28 was utilized. Quantitative data were evaluated using the Kolmogorov–Smirnov test and direct data visualization techniques, and then displayed as means and standard deviations, medians, and ranges. Numbers and percentages were used to display the numerical data. Quantitative data were compared using the Mann–Whitney (U) test or the independent *t*-test. Where appropriate, categorical data were compared using Fisher’s exact test or Chi-square analysis. To forecast positive MPI, multivariate logistic regression analysis was performed. We computed odds ratios with 95% confidence intervals. Each statistical test has two sides. Significant P values were those with a value of less than 0.05.

## Results

The current study included 443 participants with a mean age of 51 ± 9 years. The average weight was 84 ± 12 kg, and the average height was 165 ± 4 cm, resulting in a mean BMI of 30.6 ± 3.8. Approximately 42.9% of participants had diabetes mellitus, 14.4% had a positive family history, and 51% had hypertension. Regarding smoking habits, 14% of participants reported being current smokers, and 1.6% were ex-smokers. Additionally, 21.4% of participants had dyslipidemia. Menopause female patients were detected in 41.9%.

Female gender includes 266 (60%) while male represented in 177 (40%). Males demonstrated significantly higher age (55 ± 10 vs. 49 ± 9, *P* < 0.0001), weight (85 ± 11 vs. 83 ± 13, *P* = 0.006), height (166 ± 4 vs. 165 ± 4, *P* = 0.02), and smoking (35% vs. 0%, *P* < 0.001) than females. In contrast, positive family history was significantly higher in females (17.3%) than in males (10.2%) (*P* = 0.037).

There were no significant differences reported regarding BMI (*P* = 0.195), diabetes mellitus (*P* = 0.831), hypertension (*P* = 0.269), and dyslipidemia (*P* = 0.992).

Males demonstrated significantly higher abnormal resting ECG (49.2% vs. 21.1%, *P* < 0.001), resting SBP (127 ± 16 vs. 123 ± 15, *P* = 0.018), resting DBP (82 ± 11 vs. 79 ± 9, *P* = 0.006), exercise duration (median = 8.3 vs. 7.6, *P* = 0.021), METS (median = 6.7 vs. 6.4, *P* = 0.011), positive ischemic changes on ECG (36.2% vs. 20.3, *P* < 0.001), EDV (median = 101 vs. 77, *P* < 0.001), ESV (median = 38 vs. 21, *P* < 0.001), TID (median = 1 vs. 0.9, *P* < 0.001), and positive MPI (55.4% vs. 9.4%, *P* < 0.001) compared to females. Additionally, exercise stage significantly differed according to gender (*P* < 0.001), with stages IV and V being higher in males (38.4% and 5.6%) than in females (29.7% and 1.1%). Furthermore, the exercise associated symptoms differed according to gender (*P* < 0.001), with chest pain being higher in males (31.6%) than in females (13.5%). In comparison, fatigue was higher in females (32.3%) compared to males (16.4%).

In contrast, females demonstrated significantly higher heart rate (88 ± 13 vs. 83 ± 12, *P* < 0.001), MAHR (166 ± 16 vs. 153 ± 21, *P* < 0.001), MAHR% (95 ± 8 vs. 91 ± 9, *P* < 0.001), EF (67 ± 6 vs. 57 ± 15, *P* < 0.001), LHR (median = 0.32 vs. 0.45, *P* < 0.001), and diaphragmatic attenuation (36.1% vs. 22%, *P* = 0.002) than males.

### Prediction of positive MPI

Multivariate logistic regression analysis was done to predict positive MPI. The model was built clinically, including age, gender, BMI, smoking, diabetes mellitus, hypertension, family history, dyslipidemia, abnormal resting ECG, and heart rate. The significant predictors were male gender, diabetes mellitus, and abnormal resting ECG. Male gender was associated with ten times increased risk of positive MPI (OR = 10, 95% CI = 5.348–18.868, *P* < 0.001). Diabetes was associated with an increased risk of positive MPI (OR = 1.82, 95% CI = 1.052–3.148, *P* = 0.032). Abnormal resting ECG was associated with about five times increased risk of positive MPI (OR = 4.626, 95% CI = 2.647–8.086, *P* < 0.001) (Table 1).

**Table 1:** Multivariate logistic regression analysis to predict positive MPI

	OR (95% CI)	*P*-value
Age (years)	1.017 (0.987–1.047)	0.274
Male gender	10 (5.348–18.868)	< 0.001*
Body mass index	0.974 (0.908–1.044)	0.454
Current smoking	0.766 (0.371–1.584)	0.472
Diabetes	1.82 (1.052–3.148)	0.032*
Hypertension	0.947 (0.557–1.609)	0.84
Positive family history	0.541 (0.229–1.276)	0.161
Dyslipidemia	1.824 (0.943–3.529)	0.074
Abnormal resting ECG	4.626 (2.647–8.086)	< 0.001*
Heart rate	0.991 (0.971–1.013)	0.431

^*^Significant *P*-value; *OR* odds ratio; *95% CI* 95% confidence interval

### In the current study we classify our patients to positive and negative MPI

#### Positive MPI patient

As shown in Table 2**,** the mean age of MPI-positive patients was 55 ± 9. Males predominated (79.7%) more than females (20.3%). The mean weight and height were 84 ± 11 kg and 166 ± 4 cm, respectively. The mean body mass index was 30.6 ± 3.6. Half of the patients had diabetes mellitus (50.4%). Half of the females (50.4%) were menopause. Only 9.8% had a positive family history. Hypertension was reported in 56.1%. About one-third were current smokers (29.3%), and only 5.7% were ex-smokers. Dyslipidemia was reported in 27.6%.

**Table 2:** General characteristics of MPI-positive patients and according to gender

		Total (n = 123)	Males (n = 98)	Females (n = 25)	*P*-value
Age (years)	Mean ± SD	55 ± 9	56 ± 8	52 ± 10	0.039*
Weight (kg)	Mean ± SD	84 ± 11	85 ± 11	81 ± 13	0.135
Height (cm)	Mean ± SD	166 ± 4	166 ± 4	164 ± 4	0.037*
Body mass index (kg/m^2^)	Mean ± SD	30.6 ± 3.6	30.8 ± 3.4	30.1 ± 4.3	0.398
Diabetes mellitus	n (%)	62 (50.4)	46 (46.9)	16 (64)	0.128
Menopause	n (%)	13 (52)	–	13 (52)	–
Positive family history	n (%)	12 (9.8)	8 (8.2)	4 (16)	0.239
Hypertension	n (%)	69 (56.1)	55 (56.1)	14 (56)	0.991
Current smoker	n (%)	36 (29.3)	36 (36.7)	0 (0)	< 0.001*
Ex-smoker	n (%)	7 (5.7)	7 (7.1)	0 (0)	
Dyslipidemia	n (%)	34 (27.6)	25 (25.5)	9 (36)	0.295

^*^Significant *P*-value

Males demonstrated significantly higher age (56 ± 8 vs. 52 ± 10, *P* = 0.039), height (166 ± 4 vs. 164 ± 4, *P* = 0.037), and smoking (36.7% current smokers vs. 0%, *P* < 0.001) than females. No significant differences were reported regarding the remaining parameters, as shown in Table 2.

#### Clinical characteristics at rest and exercise

Two-thirds (61.8%) of MPI-positive patients demonstrated abnormal resting ECG. The mean heart rate was 84 ± 12. The mean resting systolic and diastolic blood pressure were 129 ± 15 and 82 ± 10 mmHg, respectively. The median exercise duration was 7.2 min, ranging from 2.5 to 13.7 min. The most frequent exercise stage was stage III (39.8%), followed by stage IV (27.6%), stage II (23.6%), stage I (7.4%), and stage V (1.6%). The median METS was 5.9, ranging from 1.4 to 13. The mean maximum achieved heart rate was 149 ± 18 b/m, while the mean maximum achieved heart rate percentage was 89 ± 7%. Half of the patients (51.6%) demonstrated positive ECG changes for ischemia, while one-third (36.6%) had negative changes. Only 10.6% and 1.6% showed submaximal stress test and equivocal changes, respectively. Regarding affected vessels, 52.4% had inferior affection, 47.6% had anterior affection, 36.5% had inferolateral affection, and 12.7% had anterolateral affection. Limiting chest pain was observed in 10.6% of the patients. The most frequent associated symptoms were chest pain (51.2%), followed by dyspnea (42.3%), fatigue (4.9%), and only (1.6%) had no associated symptoms. The mean systolic and diastolic blood pressures at maximum exercise were 168 ± 26 and 96 ± 12 mmHg, respectively.

Females demonstrated significantly higher maximum achieved heart rate (156 ± 12) than males (147 ± 19) (*P* = 0.036). No significant differences were reported regarding the remaining parameters as shown in Table 3.

**Table 3:** Clinical characteristics of MPI-positive patients at rest and exercise according to gender

		Total (n = 123)	Males (n = 98)	Females (n = 25)	*P*-value
Abnormal resting ECG	n (%)	76 (61.8)	62 (63.3)	14 (56)	0.505
Heart rate (b/m)	Mean ± SD	84 ± 12	83 ± 12	87 ± 11	0.102
Resting SBP (mmHg)	Mean ± SD	129 ± 15	129 ± 14	127 ± 18	0.411
Resting DBP (mmHg)	Mean ± SD	82 ± 10	83 ± 10	80 ± 10	0.260
Exercise duration (min)	Median (range)	7.2 (0.5–13.7)	7.4 (1.4–13.7)	6.5 (0.5–10.2)	0.076
Exercise stage					
Stage I	n (%)	9 (7.3)	7 (7.1)	2 (8)	0.087
Stage II	n (%)	29 (23.6)	22 (22.4)	7 (28)	
Stage III	n (%)	49 (39.8)	35 (35.7)	14 (56)	
Stage IV	n (%)	34 (27.6)	32 (32.7)	2 (8)	
Stage V	n (%)	2 (1.6)	2 (2)	0 (0)	
METS	Median (range)	5.9 (1.4–13)	6.25 (2.1–13)	5.2 (1.4–11)	0.098
MAHR (b/m)	Mean ± SD	149 ± 18	147 ± 19	156 ± 12	0.036*
MAHR (%)	Mean ± SD	89 ± 7	89 ± 8	91 ± 5	0.113
ECG changes					
Positive for ischemia	n (%)	63 (51.2)	48 (49)	15 (60)	0.324
Negative for ischemia	n (%)	45 (36.6)	37 (37.8)	8 (32)	
Equivocal	n (%)	2 (1.6)	1 (1)	1 (4)	
Submaximal stress test	n (%)	13 (10.6)	12 (12.2)	1 (4)	
Anterior affection^†^	n (%)	30 (47.6)	23 (47.9)	7 (46.7)	0.933
Inferior affection^†^	n (%)	33 (52.4)	26 (54.2)	7 (46.7)	0.612
Inferolateral affection^†^	n (%)	23 (36.5)	18 (37.5)	5 (33.3)	0.770
Anterolateral affection^†^	n (%)	8 (12.7)	6 (12.5)	2 (13.3)	0.933

^*^Significant *P*-value; †Percentages were calculated based on those with positive ischemic changes on ECG; *METS* metabolic equivalent of task; *MAHR* maximum achieved heart rate; *SBP* systolic blood pressure; *DBP* diastolic blood pressure

#### MPI findings

Approximately half (52%) of the MPI-positive patients demonstrated one defect, while the remaining (48%) had two defects. Male patients demonstrated ischemia in 88 cases; 14 cases had ischemia (total reversible defect) only and 74 cases had ischemia and scar (partially reversible) while 10 cases had scar only (fixed defect) with no ischemia. None of female patients had scar (fixed defect). Ischemia (total reversible defect) was present in 10 cases only while the remaining had both ischemia and fixed defects (partially reversible).

The median and range of ischemia was 15.5% ranging from (2 to 46) in male compared to 16% ranging from (3 to 33) in female. This difference didn’t reach to a significant difference.

The scar in totally irreversible or partially reversible ischemia was present in 84 cases in male and 15 cases in female. The median for all was 9% (range 2–67), median in male was 10% (range 2–67) and median in female was 7% and range from (2 to 33). The median SSS was 17.5%, with values ranging from (7 to 57) in male and 13% with value range from (7 to 42) in female. The median sum difference was 8% in male with a range from 1 to 32 in male. The median SDS in female was 10%, with values ranging from 0 to 32. Regarding ischemia severity, mild, moderate and severe diseases represented 35%, 19.5%, and 45.5%, respectively. The mean dose rest and stress were 28 ± 1 and 27 ± 1, respectively. The median EDV and ESV were 120 and 49, respectively. The mean EF was 52 ± 15.

The mean Lung heart ratio was 0.38 ± 0.07. The median transient ischemic dilatation was 1.19, ranging from 0.7 to 0.9. Males demonstrated significantly higher scar (median = 8 vs. 2, *P* = 0.003), EDV (median = 126 vs. 96, *P* < 0.001), and ESV (median = 59 vs. 33, *P* < 0.001) but lower ejection fraction (49 ± 16 vs. 62 ± 8, *P* < 0.001) than females. No significant differences were reported regarding the remaining parameters as shown in Table 4.

**Table 4:** Findings of positive MPI studies in all patients and according to gender

		Total (n = 123)	Males (n = 98)	Females (n = 25)	*P*-value
Numbers of defect					
One	n (%)	64 (52)	48 (49)	16 (64)	0.18
Two	n (%)	59 (48)	50 (51)	9 (36)	
Total ischemia%	Median (range)	16 (2–46)	15.5 (2–46)	16 (3–33)	0.33
Total scar%	Median (range)	9 (2–67)	10 (2–67)	7 (2–33)	**0.003**
SSS%	Median (range)	16 (7–57)	17.5 (7–57)	13 (7–42)	0.25
SRS%	Median (range)	5 (1–56)	6.5 (0–56)	2 (0–31)	0.02
SDS%	Median (range)	8 (1–32)	8 (0–32)	10 (0–32)	0.065
Ischemia severity					
Mild (SSS < 6)	n (%)	43 (35.0)	34 (34.7)	9 (36.0)	0.762
Moderate (SSS 6–10)	n (%)	24 (19.5)	18 (18.4)	6 (24.0)	
Severe (SSS > 10)	n (%)	56 (45.5)	46 (46.9)	10 (40.0)	
Dose rest	Mean ± SD	28 ± 1	28 ± 1	28 ± 2	0.629
Dose stress	Mean ± SD	27 ± 1	27 ± 1	27 ± 2	0.576
EDV (mL)	Median (range)	120 (44–413)	126 (44–413)	96 (46–160)	< 0.001*
ESV (mL)	Median (range)	49 (7–342)	59 (11–342)	33 (7–86)	< 0.001*
EF (%)	Mean ± SD	52 ± 15	49 ± 16	62 ± 8	< 0.001*
LHR	Mean ± SD	0.38 ± 0.07	0.38 ± 0.06	0.37 ± 0.1	0.770
TID	Median (range)	1.19 (0.7–9.0)	1.19 (0.7–9)	1.1 (0.8–1.3)	0.405

^*^Significant *P*-value; *EDV* end diastolic volume; *ESV* end systolic volume; *EF* ejection fraction; *LHR* lung heart ratio; *TID* transient ischemic dilatation; *SSS* sum stress score; *SRS* sum rest score; *SDS* sum difference score

### Medications

The most frequent medication used by MPI-positive patients was aspirin (70.7%), followed by beta-blockers (43.9%), anti-lipids (36.6%), ACEI (36.6%), Plavix (35.8), nitroglycerin (32.5%), ARBs (5.7%), and CCB (4.1%).

Males demonstrated significantly higher use of nitroglycerin (36.7% vs. 16%, *P* = 0.048) and aspirin (75.5% vs. 52%, *P* = 0.021). No significant differences were observed regarding the remaining drugs according to gender as shown in Table 5.

**Table 5:** Medications used in MPI-positive patients according to gender

		Total (n = 123)	Males (n = 98)	Females (n = 25)	*P*-value
Beta blockers	n (%)	54 (43.9)	47 (48)	7 (28)	0.073
Nitroglycerin	n (%)	40 (32.5)	36 (36.7)	4 (16)	0.048*
Aspirin	n (%)	87 (70.7)	74 (75.5)	13 (52)	0.021*
Plavix	n (%)	44 (35.8)	35 (35.7)	9 (36)	0.979
Anti-lipids	n (%)	45 (36.6)	35 (35.7)	10 (40)	0.691
ACEI	n (%)	45 (36.6)	40 (40.8)	5 (20)	0.054
ARBs	n (%)	7 (5.7)	5 (5.1)	2 (8)	0.577
CCB	n (%)	5 (4.1)	4 (4.1)	1 (4)	1.0

^*^Significant *P*-value; *ACEI* angiotensin-converting enzyme inhibitors; *ARBs* angiotensin II receptor blockers; *CCB* calcium channel blockers

## Discussion

Females represented around 60% of our patients included in this study. This percentage and numbers are much higher than represented in many other MPI studies with male predominance. This related to the culture in Egypt and Middle East that female patients and their families prefer female physicians for their medical care and managements as our hospital belong to a faculty of medicine for girls.

Despite the presence of typical chest pain and unexplained dyspnea and fatigue to refer patients for MPI study; only 9.4% of female patients had a positive MPI study while the percentage was 55.4% in male patients. However, the age of female patients with positive test was younger than male patients significantly.

Moreover, our female patient’s age was younger than age reported in Cerci et al. study. The mean age of their study was 64.5 ± 5.6 years (range from 55 to 75 years) [12].

The reasons of ischemia of female in young age may be related to the presence of multiple risk factors in our study population as all females were over-weighted to obese, 64% of them were diabetics, around half of them were menopause and more than one third of them were dyslipidemic.

Recently, other risk factors were added in women including factors related to pregnancy, hormonal factors, social factors (race, education, income and zip code) and psychological risk factors [13].

Though this difference is not constant across all nations, men had a higher age-standardized CVD death rate globally in 2019 (280.8 deaths per 100,000 people) than women (204.0 deaths per 100,000 people). In the Middle East and North Africa, Egypt has the most disparities, with 600.0 against 491.6 fatalities per 100,000 inhabitants for women and men, respectively [14].

Female patients with negative MPI test represent higher percentage despite their symptoms including typical chest pain. They are younger in age (mean ± SD was 48.4 ± 8.4 years) but the reason(s) for this result will be questionable and require explanation. Did the test revealed false negative result as published before? Is chest pain related to coronary spasm or psychological risk factors?

There is significant difference between male and female in scar size, EDV, ESV and EF and no significant difference regarding SSS, SRS, SD, severity of ischemia. Reviewing other literature, female had less severe ischemia than men [15] but in this study the author include 4 different stress testing modalities which increases generalizability of his result.

Male patients revealed a higher percentage (55.4%) of the positive MPI test from all males participated in this study. Multivariate logistic regression demonstrated that male gender was associated with ten times increased risk of positive MPI.

Fixed Irreversible ischemia was significantly higher in males (*p* < 0.003) and about 10% of positive male cases had scar only with no reversible ischemia.

Males are believed to have more ischemic heart disease than females especially before menopause and as females aging the risk for development of ischemic heart disease is increasing [16].

We found in our study that a positive family history was more common in females than in males referred to MPI study; however, it was not predictive of a positive MPI result. Although Bachmann et al. 2012 reported that family history was associated with increased risk for future cardiovascular events whatever it was premature or late [17]. This was also confirmed by another study by Chacko et al. [18] who stated that positive family history is an independent risk factor for premature coronary artery disease and that the risk increases by the increase of number of family members affected.

The mean exercise duration was more in males than females regardless the MPI test results, it was lower in both males and females in positive group with lesser duration in females, this was concordant with previous finding reported by Wu et al. [19]. This may be attributed to the low functional capacity in females than males [20].

Although the prevalence of diabetes mellitus in our study population was nearly similar between females and males, the presence of DM was associated with an 82% increased risk of positive MPI. DM is a well-established risk factor for development of ischemic heart disease and considered as coronary artery disease equivalent. DM affects both epicardial coronary arteries and microcirculation [21].

## Limitations

This study has some limitations. The small number of positive female cases and lack of similar study to compare with in the Egyptian population. Females referred to cardiac SPECT-MPI with negative result may have IHD and the coronary angiography remains the gold standard for diagnosis of CAD but an invasive technique.

The final SPECT-MPI result was interpreted by expertise instead of blind interpretations.

## Conclusions

Positive MPI test are more common in males. Female patients with positive MPI were younger than male patients in this study and that in contrary to what documented in literatures before. Diabetes mellitus and age are traditional strong predictors for the presence of positive MPI test. Presence of scar was exclusive in male. However, to confirm or exclude the presence of IHD in symptomatic females with negative MPI, it still requires furthermore affordable non–invasive method(s) to clarify the link between symptoms and ischemia in women.

## Data Availability

The datasets used and/or analyzed during the current study are available from the corresponding author on reasonable request.

## Abbreviations

CAD: Coronary artery disease
DM: Diabetes mellitus
IHD: Ischemic heart disease
MPI: Myocardial perfusion imaging
